# Medical nutrition therapy and clinical outcomes in critically ill adults: a European multinational, prospective observational cohort study (EuroPN)

**DOI:** 10.1186/s13054-022-03997-z

**Published:** 2022-05-18

**Authors:** Martin Matejovic, Olivier Huet, Karolien Dams, Gunnar Elke, Clara Vaquerizo Alonso, Akos Csomos, Łukasz J. Krzych, Romano Tetamo, Zudin Puthucheary, Olav Rooyackers, Inga Tjäder, Helmut Kuechenhoff, Wolfgang H. Hartl, Michael Hiesmayr

**Affiliations:** 1 First Medical Department, Faculty of Medicine in Pilsen, Charles University and University Hospital in Pilsen, Pilsen, Czech Republic; 2 CHRU La Cavale Blanche, Brest, France; 3 Department of Critical Care Medicine, Antwerp University Hospital, University of Antwerp, Edegem, Belgium; 4 Department of Anesthesiology and Intensive Care Medicine, University Medical Center Schleswig-Holstein, Campus Kiel, Kiel, Germany; 5 Department of Intensive Care Medicine, Fuenlabrada University Hospital (Hospital Universitario de Fuenlabrada), Madrid, Spain; 6 MH EK Honvedkorhaz, Budapest, Hungary; 7 Department of Anesthesiology and Intensive Care, Medical University of Silesia, Katowice, Poland; 8 Ospedale AUSL Reggio Emilia, Reggio Emilia, Italy; 9 Barts Health (Royal London) and Queen Mary University of London, London, England, UK; 10 Division of Anesthesiology and Intensive Care, Department of Clinical Science, Intervention and Technology, Karolinska Institutet, Stockholm, Sweden; 11 Karolinska University Hospital, Perioperative Medicine and Intensive Care, Huddinge, Stockholm, Sweden; 12 Statistisches Beratungslabor, Institut für Statistik Ludwig-Maximilians-Universität München, Munich, Germany; 13 Klinik für Allgemeine, Viszeral- und Transplantationschirurgie, Klinikum der Universität, Campus Grosshadern, Ludwig-Maximilians-Universität München, Marchioninistraße 15, 81377 Munich, Germany; 14 Division of Cardiac, Thoracic, Vascular Anesthesia and Intensive Care, and Center for Medical Statistics, Informatics and Intelligent Systems, Medical University Vienna, Spitalgasse 23, Vienna, Austria

**Keywords:** Critical illness, Mechanical ventilation, Weaning, Survival, Nutrition, Calorie, Protein

## Abstract

**Background:**

Medical nutrition therapy may be associated with clinical outcomes in critically ill patients with prolonged intensive care unit (ICU) stay. We wanted to assess nutrition practices in European intensive care units (ICU) and their importance for clinical outcomes.

**Methods:**

Prospective multinational cohort study in patients staying in ICU ≥ 5 days with outcome recorded until day 90. Macronutrient intake from enteral and parenteral nutrition and non-nutritional sources during the first 15 days after ICU admission was compared with targets recommended by ESPEN guidelines. We modeled associations between three categories of daily calorie and protein intake (low: < 10 kcal/kg, < 0.8 g/kg; moderate: 10–20 kcal/kg, 0.8–1.2 g/kg, high: > 20 kcal/kg; > 1.2 g/kg) and the time-varying hazard rates of 90-day mortality or successful weaning from invasive mechanical ventilation (IMV).

**Results:**

A total of 1172 patients with median [Q1;Q3] APACHE II score of 18.5 [13.0;26.0] were included, and 24% died within 90 days. Median length of ICU stay was 10.0 [7.0;16.0] days, and 74% of patients could be weaned from invasive mechanical ventilation. Patients reached on average 83% [59;107] and 65% [41;91] of ESPEN calorie and protein recommended targets, respectively. Whereas specific reasons for ICU admission (especially respiratory diseases requiring IMV) were associated with higher intakes (estimate 2.43 [95% CI: 1.60;3.25] for calorie intake, 0.14 [0.09;0.20] for protein intake), a lack of nutrition on the preceding day was associated with lower calorie and protein intakes (− 2.74 [− 3.28; − 2.21] and − 0.12 [− 0.15; − 0.09], respectively). Compared to a lower intake, a daily moderate intake was associated with higher probability of successful weaning (for calories: maximum HR 4.59 [95% CI: 1.5;14.09] on day 12; for protein: maximum HR 2.60 [1.09;6.23] on day 12), and with a lower hazard of death (for calories only: minimum HR 0.15, [0.05;0.39] on day 19). There was no evidence that a high calorie or protein intake was associated with further outcome improvements.

**Conclusions:**

Calorie intake was mainly provided according to the targets recommended by the active ESPEN guideline, but protein intake was lower. In patients staying in ICU ≥ 5 days, early moderate daily calorie and protein intakes were associated with improved clinical outcomes.

*Trial registration* NCT04143503, registered on October 25, 2019.

**Graphical abstract:**

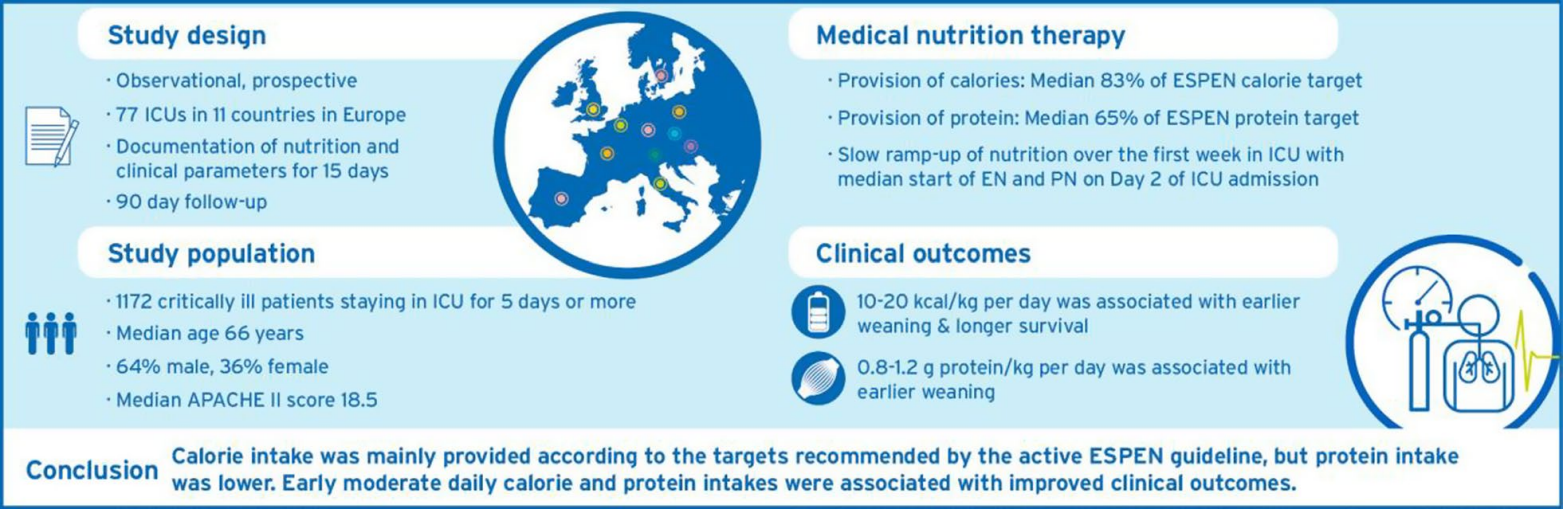

**Supplementary Information:**

The online version contains supplementary material available at 10.1186/s13054-022-03997-z.

## Background

Medical nutrition therapy is an integral part of critical care. The current European Society for Clinical Nutrition and Metabolism (ESPEN) critical care guidelines recommend a progressive ramp-up of medical nutrition therapy providing < 70% of measured energy expenditure or of estimated needs during the early phase of acute illness, and up to 80–100% after day three, to limit the risk of overfeeding and refeeding syndrome [1]. In parallel, 1.3 g/kg protein equivalents per day can be delivered progressively. These recommendations are more conservative than those propagated by the preceding ESPEN guideline [2] acknowledging evidence of a detrimental effect of high calorie intakes during the acute phase, and that a higher protein intake during the first week of critical illness may improve clinical outcomes [3].

The majority of critically ill patients do not receive adequate nutritional intake according to guideline targets [4, 5]. Currently, there is no study assessing the level of adherence to the new ESPEN recommendations. The evidence from RCTs and observational studies on the specific amounts of calories and protein and the timing of medical nutrition therapy and its relation to clinical outcomes, e.g., weaning from invasive mechanical ventilation (IMV) and survival [6–14], are inconsistent, and few studies have focused on long-stay ICU patients. Consequently, there is also a strong need to re-assess the importance of macronutrient intake for clinical outcomes.

The aim of the present study was to describe medical nutrition therapy for up to 15 days after ICU admission in European critically ill patients with a minimum length of stay (LOS) of 5 days and to assess daily associations between calorie and protein intake with time to weaning from invasive mechanical ventilation, and 90-day survival time.

## Methods

### Study design

The present study was a multinational, prospective observational cohort study conducted between November 2019 and July 2020 in 11 European countries (Austria, Belgium, Czech Republic, France, Germany, Hungary, Italy, Poland, Spain, Sweden, and the UK), approved by the respective Ethics Committees and Institutional Review Boards. The study protocol was published previously [15].

### Patients

The study included critically ill adults, aged 18–95 years, with a body mass index (BMI) of ≥ 18.5 kg/m^2^ and ≤ 45 kg/m^2^, hospitalized in any type of ICUs for at least five consecutive days. Exclusion criteria included: burns; chronic, preexisting neuromuscular, psychiatric, or neurological conditions precluding assessment of functional status; home nutritional support or chronic mechanical ventilation before or at the time of ICU admission; palliative care; or concurrent enrolment in any nutrition-related interventional study. After informed consent, demographic characteristics were recorded at ICU admission and clinical, and nutrition variables daily until ICU discharge, death, or maximum day 15 of ICU stay. Outcome was recorded until day 90 either by visit if hospitalized or via telephone if already discharged. Patient’s mobility status (ICU mobility score [16], IMS) was estimated at baseline, reflecting IMS values before ICU admission, and observed at day 15, 30, and 90 after ICU admission.

### Outcomes

The primary outcome of this study was described as the median [Q1;Q3] calorie and protein balances, calculated as the percentage deviation from the ESPEN targets until ICU discharge, death, or maximum day 15 of ICU stay. Intakes were calculated from all nutritional sources, i.e., oral nutrition, oral nutritional supplements (ONS), enteral nutrition (EN), parenteral nutrition (PN), and non-nutritional calories, i.e., propofol, clevidipine, citrate, or glucose. ESPEN guidelines recommend a progressive increase in calorie intake until an estimated energy expenditure of 25 kcal/kg per day. We defined the progressive increase as 10 kcal/kg body weight (BW) on day 1 (≈ 40% of energy expenditure), 15 kcal/kg on day 2 and 3 (≈ 60% of energy expenditure), 20 kcal/kg on day 4 to 6 (≈ 80% of energy expenditure), 25 kcal/kg on day 7 to 15 (≈ 100% of energy expenditure).

Daily protein targets were set at 0.6 g/kg on day 1, 0.9 g/kg on day 2 and 3, and 1.3 g/kg on days 4 to 15. Calculations were based on actual (admission) BW for patients with a BMI < 30 kg/m^2^ and on adjusted BW, determined by the formula: (actual BW—ideal BW) × 0.33 + ideal BW when BMI was ≥ 30 kg/m^2^, estimating ideal BW as per Peterson [17].

Other outcomes were time to weaning from IMV, defined as the time in days from the start of IMV to either successful weaning (irrespective of subsequent death) or to death while intubated, and 90-day survival time.

### Quantifying macronutrient intake

Total daily protein intake was classified by using established thresholds [18] defining three different levels based on the daily amount of received protein (level I, low: < 0.8 g protein/kg; level II, moderate: 0.8–1.2 g protein/kg; level III, high: > 1.2 g protein/kg).

Total daily calorie intake was also classified into three levels: [19–21]: level I, low: < 10 kcal/kg per day; level II, moderate: 10–20 kcal/kg per day; level III, high: > 20 kcal/kg per day.

### Statistical analyses

To facilitate interpretation of the time-varying hazard ratios (HR) between nutrition and outcome, we predefined different hypothetical time-varying medical nutrition therapies with three levels of calorie or protein intake (low, moderate, high) in combination with an early (day 1 to 4) and late (after day 4) period over days 1–15 (Table 1). Importantly, these hypothetical medical nutrition therapies represent concepts similar to clinically established nutrition protocols, but do not reflect selected patient cohorts contained in this study. We modeled associations between these hypothetical medical nutrition therapies and outcomes by designing six pairwise medical nutrition therapy comparisons while controlling for confounders. All hazard ratios of these pairwise comparisons of different hypothetical medical nutrition therapies were calculated under the assumption that all other variables were fixed and are presented in the results as maximum/minimum HR at a given day, with 95% CI.

**Table 1:** Description of hypothetical medical nutrition therapies

Medical nutrition therapy	Description
*Early*	*Feeding on days #1 to #4*
Low,	< 10 kcal/kg per day, or < 0.8 g protein/kg per day
Moderate, or	10–20 kcal/kg per day, or 0.8–1.2 g protein/kg per day
High calorie/protein intake	> 20 kcal/kg per day, or > 1.2 g protein/kg per day
*Late*	*Feeding on days #5 to #15*
Low	< 10 kcal/kg per day, or < 0.8 g protein/kg per day,
Moderate, or	10–20 kcal/kg per day, or 0.8–1.2 g protein/kg per day
High calorie/protein intake	> 20 kcal/kg per day, or > 1.2 g protein/kg per day
*Exclusively*	*Feeding on days #1 to #15*
Low	< 10 kcal/kg per day, or < 0.8 g protein/kg per day
Moderate, or	10–20 kcal/kg per day, or 0.8–1.2 g protein/kg per day
High calorie/protein intake	> 20 kcal/kg per day, or > 1.2 g protein/kg per day

Number of days with a defined level of medical nutrition therapy starts with the day of ICU admission (day #1)

For survival analysis, our statistical model required information on calorie and protein intake for all 15 days after ICU admission, even if a patient had been discharged from the ICU before. We, therefore, imputed a daily calorie and protein intake reflecting 80% of our patient`s average preceding intake of the three last days prior to discharge (on average, 1.2 g protein/kg per day and 20 kcal/kg per day); no other imputations were performed (e.g., for the time to weaning from IMV analysis and when analyzing predictors of macronutrient intake).

A detailed explanation of the methodology and how to translate these pairwise comparisons into the cox-type models is provided in Additional file 1: Annex 1, Additional file 1: Fig. S1.

## Results

### Study population

**Fig. 1:**
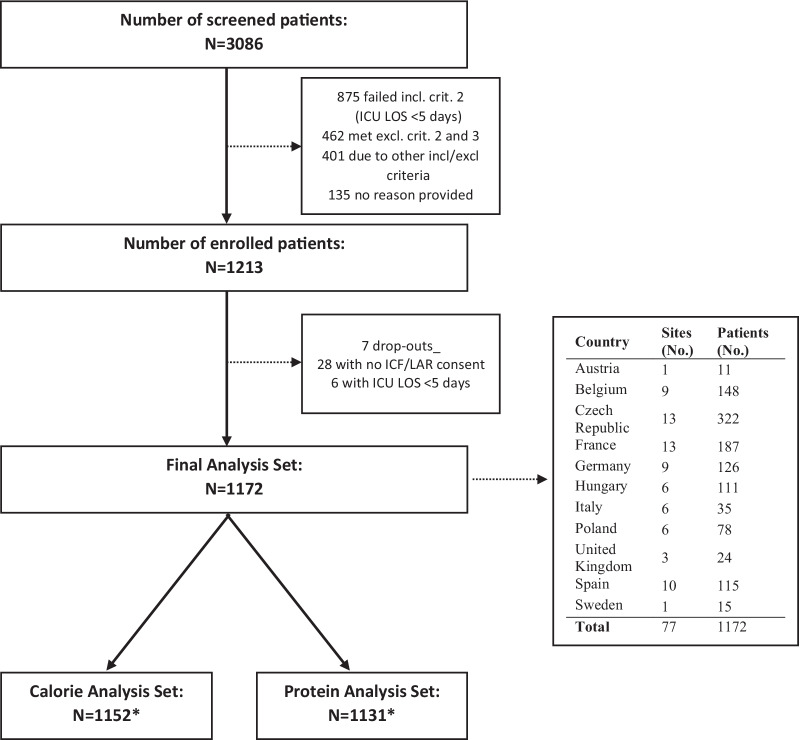
Flowchart. *No values on calorie and protein intake were provided for 20 and 41 patients who received oral nutrition only. *ICF* Informed consent form, *LAR* legal representative

A total of 3086 patients were screened in 77 ICUs from 11 countries. A total of 1213 patients were enrolled, of whom 34 were excluded, and 7 dropped out, while 1172 underwent analysis (Fig. 1). Patient characteristics are shown in Table 2. By day 15, 344 (29%) patients were still in ICU, 474 (40%) still hospitalized, and 352 (30%) had been discharged home or transferred to another healthcare facility, with 2 patients (0.2%) lost to follow-up. By day 90, 71 (6%) were still in hospital or ICU. Regarding mortality, 95 (8%) and 275 (24%) patients had died by day 15 and day 90, respectively (Kaplan–Meier and cumulative incidence plots in Additional file 1: Annex 1, Additional file 1: Fig. S2, Fig. S3).

**Table 2:** Demographic, nutrition, clinical, and follow-up characteristics of the study population

Characteristic (*n* = 1172^a^)	No. (%) or median [Q1;Q3]
Age, years	66.0 [56.0;74.0]
Sex	
Male	745 (63.6)
Female	427 (36.4)
BMI, kg/m^2^, *n* = 1168	26.8 [24.0;31.1]
APACHE II score at ICU admission, *n* = 1132	18.5 [13.0;26.0]
SOFA score at ICU admission, *n* = 1042	7.0 [4.0;10.0]
Number of comorbidities at ICU admission	3.0 [1.0;5.0]
Type of ICU admission	
Non-surgical emergency	573 (48.9)
Surgical emergency	360 (30.7)
Surgical elective	220 (18.8)
Others	19 (1.6)
Main reason(s) for ICU admission^b^	
Respiratory	546 (46.6)
Infection	362 (30.9)
Cardiac	343 (29.3)
Hepatic/GI/digestive	279 (23.8)
Neurological	134 (11.4)
Renal	126 (10.8)
Trauma	116 (9.9)
Others	74 (6.3)
Sedation^c^	885 (75.5)
Sum of days on sedation, *n* = 885	5.0 [2.0;10.0]
Vasopressors^c^	886 (75.6)
Sum of days on vasopressors, *n* = 886	4.0 [2.0;8.0]
Ventilatory support^c,d^	988 (84.3)
Sum of days on ventilatory support, *n* = 988	7.0 [4.0;13.0]
Invasive mechanical ventilation between day 1 to 3 of ICU admission	813 (69.4)
Physiotherapy^c^	1006 (85.8)
Sum of days on physiotherapy, *n* = 1006	7.0 [4.0;11.0]
Healthcare-associated infections after day 3 of ICU admission	230 (19.6)
Days in ICU until first infection, *n* = 230	6.0 [4.0;9.0]
Mobility status (IMS score)	10.0 [8.0;10.0]
before ICU admission, *n* = 1168	10.0 [8.0;10.0]
At day 15, *n* = 1057	5.0 [1.0;10.0]
At day 30, *n* = 949	9.0 [3.0;10.0]
At day 90, *n* = 844	10.0 [9.0;10.0]
ICU LOS, days, *n* = 1158	10.0 [7.0;16.0]
Hospital LOS, days, *n* = 1077	23.0 [15.0;36.0]
ICU mortality, *n* = 1082	168 (15.5)
Hospital mortality, *n* = 1082	244 (22.6)
90-day mortality, *n* = 1172	276 (23.5)

*APACHE* acute physiology and chronic health evaluation, *BMI* body mass index, *ICU* intensive care unit, *IMS* ICU mobility scale, *IQR* interquartile range, *LOS* length of stay, *SOFA* Sequential Organ Failure Assessment

^a^Unless otherwise indicated

^b^Multiresponse variable

^c^On at least one day during the first 15 observation days

^d^Includes invasive, noninvasive ventilation and high flow nasal oxygen therapy

Half of the 813 patients requiring IMV between day 1 and 3 after ICU admission were weaned by day 8. A total of 601 (74%) weaning events occurred during the 15-day observation period (cumulative incidence plot in Additional file 1: Annex 1, Additional file 1: Fig. S3).

### Calorie and protein intake

**Fig. 2:**
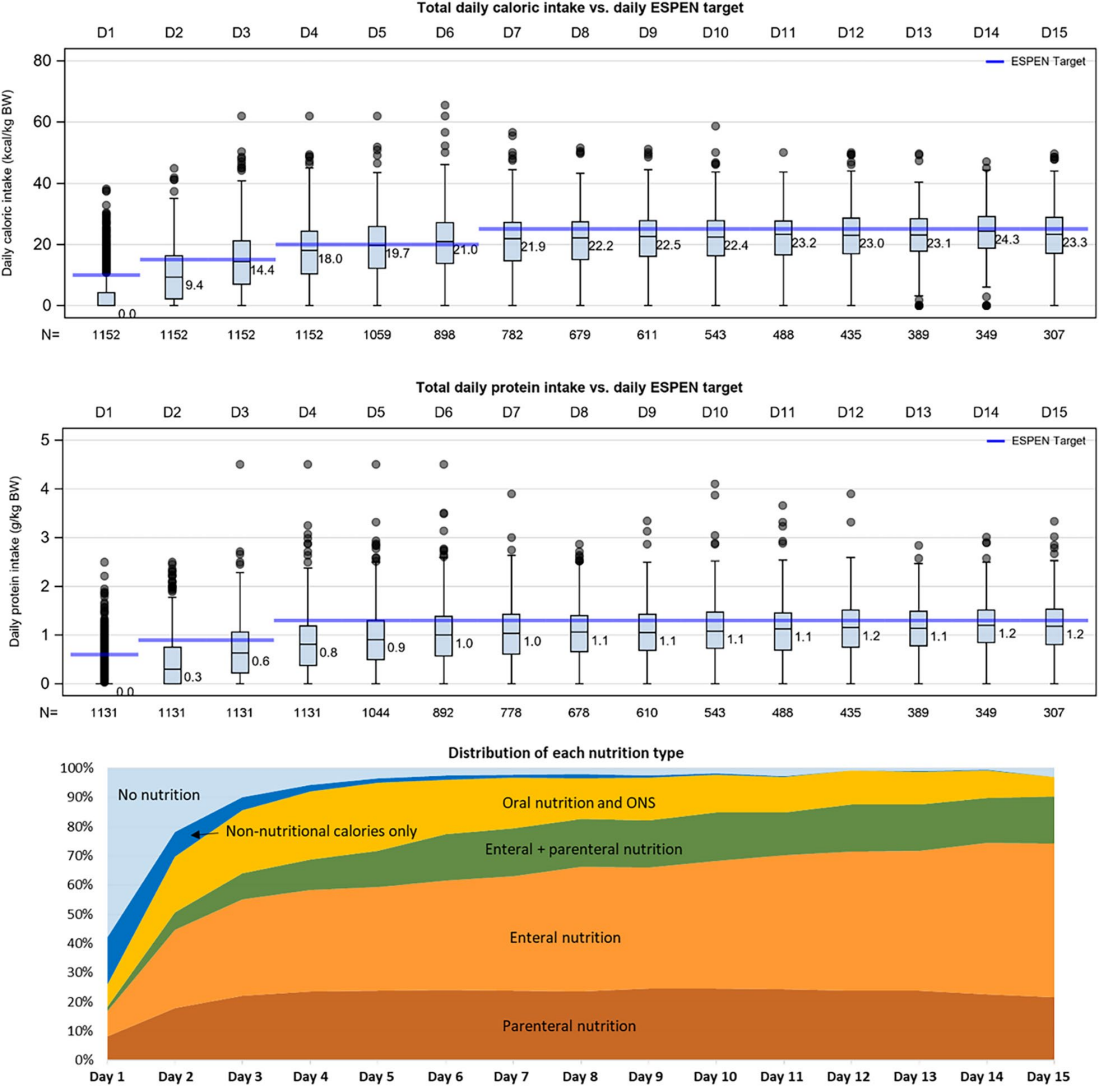
Daily calorie and protein intake, and distribution of nutrition resources. Intake is presented as median, interquartile range, minimum, and maximum values with outliers versus pre-defined targets (blue horizontal bars) based on the 2019 ESPEN Guideline on Clinical Nutrition in Critical Care, and with proportion of nutrition resources used on a respective day. ESPEN-defined daily calorie intake targets were 10 kcal/kg on D1, 15 kcal/kg on D2–D3, 20 kcal/kg on D4–D6, 25 kcal/kg on D7–D15. ESPEN-defined daily protein intake targets were 0.6 g/kg on D1, 0.9 g/kg on D2–D3, 1.3 g/kg on D4–D15. Non-nutritional calories included the use of glucose solutions, propofol, clevidipine, and citrate from renal replacement therapy. Patients without any nutrition on a respective day were counted with 0 kcal or 0 g protein. The EN and/or PN categories also included patients who had received small amounts of calories/protein from oral nutrition/ONS. *EN* enteral nutrition, *ON* oral nutrition, *ONS* oral nutritional supplements, *PN* parenteral nutrition

Nutrition started on median ICU day 2.0 [2.0;4.0] for PN, 2.0 [2.0;4.0] for EN and 3.0 [2.0;6.0] for oral nutrition/ONS. The provision of calories and proteins increased progressively over the first 5 days from a median intake of 0.0 kcal/kg and 0.0 g/kg protein on day 1, because most patients were not nourished on this day, to 19.7 kcal/kg and 0.9 g/kg protein on day 5 (Fig. 2). This ramp-up was also present when patients that had not received any nutrition on a specific day were excluded. Daily intake in this subgroup increased from a median of 5.6 kcal/kg and 0.5 g/kg protein on day 1, to 20.2 kcal/kg and 1.0 g/kg protein on day 5 (Additional file 1: Annex 1, Additional file 1: Fig. S4, Fig. S5). On day 3 of ICU admission, combined enteral/oral calorie intake was on average 8 kcal/kg per day, and increased to about 13 kcal/kg per day between days 6 and 14. Average parenteral calorie intake was 5 kcal/kg per day on day 3, and increased to about 7 kcal/kg per day between days 6 and 14.

Across all study days, median daily calorie and protein intake was 15.9 [10.8;2.2] kcal/kg and 0.7 [0.4;1.0] g/kg, respectively. Patients met on average 83% [59;107] of the calorie and 65% [41;91] of the ESPEN protein targets over the study period. The amount of information present in each of our nutrition levels (i.e., low, moderate, high) was well balanced, and the median daily calorie and protein intake increased from the low to the high level (Table 3).

**Table 3:** Number of days with a specific intake level and median macronutrient intake of each level

Calorie and protein levels	Days (%)	Median [95% CI]
Total days with information on calorie intake: 11,152		
Low, < 10 kcal/kg	3089 days (27.7%)	2.3 [0.0;6.5] kcal/kg
Moderate, 10–20 kcal/kg	3187 days (28.6%)	15.5 [12.8;18.0] kcal/kg
High, > 20 kcal/kg	4876 days (43.7%)	26.3 [23.1;30.3] kcal/kg
Total days with information on protein intake: 11,038		
Low, < 0.8 g/kg	5300 days (48.0%)	0.28 [0.0;0.55] g/kg
Moderate, 0.8–1.2 g/kg	2599 days (23.5%)	1.0 [0.90;1.11] g/kg
High, > 1.2 g/kg	3139 days (28.5%)	1.51 [1.36;1.76] g/kg

A lower calorie and protein intake was significantly predicted by a lack of nutrition on the preceding day (estimate for calorie intake − 2.74, 95% CI [− 3.28; − 2.21]; estimate for protein intake − 0.12 [− 0.15; − 0.09]), and by a regular assessment of individual nutritional needs, performed in 73 (95%) of the 77 participating ICUs (estimate for calorie intake − 2.86 [− 4.44; − 1.28]; estimate for protein intake − 0.22 [− 0.32; − 0.12]), whereas a higher calorie and protein intake was predicted by the need for IMV on the preceding day (estimate for calorie intake 2.43 [95% CI: 1.60;3.25]; estimate for protein intake 0.14 [0.09;0.20]), by a respiratory reason for ICU admission (estimate for calorie intake 2.43 [1.60; 3.25]; estimate for protein intake 0.14 [0.09; 0.20]) and by a hepatic reason (estimate for calorie intake 2.29 [1.36;3.23]; estimate for protein intake 0.13 [0.07;0.18]) (Tables 4, 5).

**Table 4:** Linear mixed effect model with repeated measures describing predictors of daily calorie intake (kcal/kg BW)

	Variable name	Estimate	LL 95% CI	UL 95% CI	*p* value
Predictor variables	Surgical versus non-surgical ICU admission	0.136	− 0.716	0.989	0.754
	Average SOFA score of preceding days	0.021	− 0.061	0.103	0.613
	Patient not nourished the preceding day (yes vs. no)	− 2.744	− 3.281	− 2.206	< 0.001
	Main reason for ICU admission: respiratory (yes vs.no)	2.426	1.604	3.249	< 0.001
	Invasive mechanical ventilation on the preceding day (yes vs. no)	1.164	0.716	1.612	< 0.001
	Nutritional needs regularly assessed for patients (yes vs. no)	− 2.861	− 4.442	− 1.279	< 0.001
	Female vs. male	1.594	0.819	2.368	< 0.001
	Main reason for ICU admission: hematologic (yes vs.no)	3.955	1.720	6.191	< 0.001
	Main reason for ICU admission: hepatic (yes vs.no)	2.293	1.357	3.229	< 0.001
Smooth terms	Age	–	–	–	0.408
	APACHE II	–	–	–	0.551
Time variable	*Analysis visit day*				
	Day 3	0.197	0.171	0.222	< 0.001
	Day 4	0.338	0.306	0.371	< 0.001
	Day 5	0.425	0.388	0.462	< 0.001
	Day 6	0.493	0.453	0.533	< 0.001
	Day 7	0.509	0.467	0.552	< 0.001
	Day 8	0.505	0.460	0.550	< 0.001
	Day 9	0.521	0.474	0.569	< 0.001
	Day 10	0.544	0.495	0.593	< 0.001
	Day 11	0.548	0.497	0.599	< 0.001
	Day 12	0.579	0.526	0.632	< 0.001
	Day 13	0.569	0.513	0.624	< 0.001
	Day 14	0.607	0.549	0.664	< 0.001
	Day 15	0.588	0.527	0.648	< 0.001

Analysis was also adjusted for study site as random effect

APACHE: Acute Physiology and Chronic Health Evaluation; ICU: intensive care unit; LL: lower limit; UL: upper limit; and CI: confidence interval. Continuous variables were modeled by flexible penalized spline to account for possible nonlinear (smoothed) relationships with the outcome variables. An estimate > 0 indicates that the variable was associated with a higher caloric intake

**Table 5:** Linear mixed effect model with repeated measures describing predictors of daily protein intake (g/kg BW)

	Variable name	Estimate	LL 95% CI	UL 95% CI	*p* value
Predictor variables	Surgical versus non-surgical ICU admission	0.033	− 0.021	0.087	0.234
	Average SOFA score of preceding days	0.003	− 0.002	0.008	0.246
	Patient not nourished the preceding day (yes vs. no)	− 0.121	− 0.150	− 0.091	< 0.001
	Invasive mechanical ventilation on the preceding day (yes vs. no)	0.083	0.058	0.108	< 0.001
	Main reason for ICU admission: Respiratory (yes vs. no)	0.143	0.091	0.195	< 0.001
	Nutritional needs regularly assessed for patients (yes vs. no)	− 0.219	− 0.319	− 0.120	< 0.001
	Main reason for ICU admission: Hepatic (yes vs. no)	0.125	0.066	0.184	< 0.001
Smooth terms	Age	–	–	–	0.422
	APACHE II	–	–	–	0.539
Time variable	*Analysis visit day*				
	Day 3	0.197	0.171	0.222	< 0.001
	Day 4	0.338	0.306	0.371	< 0.001
	Day 5	0.425	0.388	0.462	< 0.001
	Day 6	0.493	0.453	0.533	< 0.001
	Day 7	0.509	0.467	0.552	< 0.001
	Day 8	0.505	0.460	0.550	< 0.001
	Day 9	0.521	0.474	0.569	< 0.001
	Day 10	0.544	0.495	0.593	< 0.001
	Day 11	0.548	0.497	0.599	< 0.001
	Day 12	0.579	0.526	0.632	< 0.001
	Day 13	0.569	0.513	0.624	< 0.001
	Day 14	0.607	0.549	0.664	< 0.001
	Day 15	0.588	0.527	0.648	< 0.001

Analysis was also adjusted for study site as random effect. APACHE: Acute Physiology and Chronic Health Evaluation; ICU: intensive care unit; LL: lower limit; UL: upper limit; and CI: confidence interval. Continuous variables were modeled by flexible penalized spline to account for possible nonlinear (smoothed) relationships with the outcome variables

An estimate > 0 indicates that the variable was associated with a higher protein intake

### Nutrition and outcome

The associations of hypothetical time-varying calorie and protein intakes with time to weaning from IMV and with survival were adjusted for main confounders (Tables 6, 7, Additional file 1: Annex 1, Additional file 1: Tables S1, S2). Percentage of total calories given via the enteral and/or oral route between day 1 and day 5 after admission was not significantly associated with survival time and time until successful weaning.

**Table 6:** Association between confounders and time to weaning from invasive mechanical ventilation (cox-type model for calorie intake)

	Variable name	HR	LL CI 95%	UL CI 95%	*p* value
Predictor variables	Age	1.00	0.99	1.00	0.245
	Female vs. male	1.23	0.98	1.55	0.079
	Body weight (kg)	1.00	0.99	1.00	0.189
	Surgical versus non-surgical ICU admission	1.45	1.12	1.90	0.006
	Main reason for ICU admission: Infection (yes vs. no)	1.25	0.99	1.59	0.066
	Main reason for ICU admission: Respiratory (yes vs. no)	0.59	0.46	0.75	< 0.001
	Number of severe comorbidities	1.13	1.00	1.28	0.046
	APACHE II	1.00	0.99	1.01	0.847
	Average SOFA score of preceding days (d1–d5)	0.89	0.85	0.92	< 0.001
	Sum of preceding days with HAIs (d1–d5)	0.90	0.83	0.97	0.006
	Baseline functional status	0.97	0.91	1.03	0.316
	% of total calories given via the enteral and oral route (d1–d5)	1.00	1.00	1.01	0.075
Random effect	Study site				< 0.001

A HR < 1 indicates a longer time until extubation

*APACHE* Acute Physiology and Chronic Health Evaluation, *ICU* intensive care unit, *HR* hazard ratio, *LL* lower limit, *UL* upper Limit, *CI* confidence interval, *HAI* hospital acquired infection

**Table 7:** Association between confounders and 90-day survival time (cox-type model for calorie intake)

	Variable name	HR	LL CI 95%	UL CI 95%	*p* value
Predictor variables	Age	1.04	1.03	1.05	< 0.001
	Body weight (kg)	0.99	0.98	1.00	0.048
	Surgical versus non-surgical ICU admission	0.62	0.45	0.86	0.004
	Main reason for ICU admission: Respiratory (yes vs. no)	1.36	1.02	1.80	0.035
	APACHE II	1.00	0.99	1.02	0.832
	Average SOFA score of preceding days (d1–d5)	1.15	1.10	1.20	< 0.001
	Sum of preceding days with HAIs (d1–d5)	1.04	1.02	1.07	0.002
	Sum of preceding days with delirium (d1–d5)	0.97	0.92	1.01	0.169
	Sum of preceding days on invasive mechanical ventilation (d1–d5)	1.01	0.98	1.03	0.663
	Limited medical support (yes vs. no)	3.91	2.28	6.72	< 0.001
	% of total calories given via the enteral and oral route (d1–d5)	1.00	0.99	1.00	0.095
Random effect	Study site				0.002

A HR > 1 indicates a shorter survival time

*APACHE* acute physiology and chronic health evaluation, *ICU* intensive care unit, *HR* hazard ratio, *LL* lower limit, *UL* upper limit, *CI* confidence interval, *HAI* hospital acquired infection

**Fig. 3:**
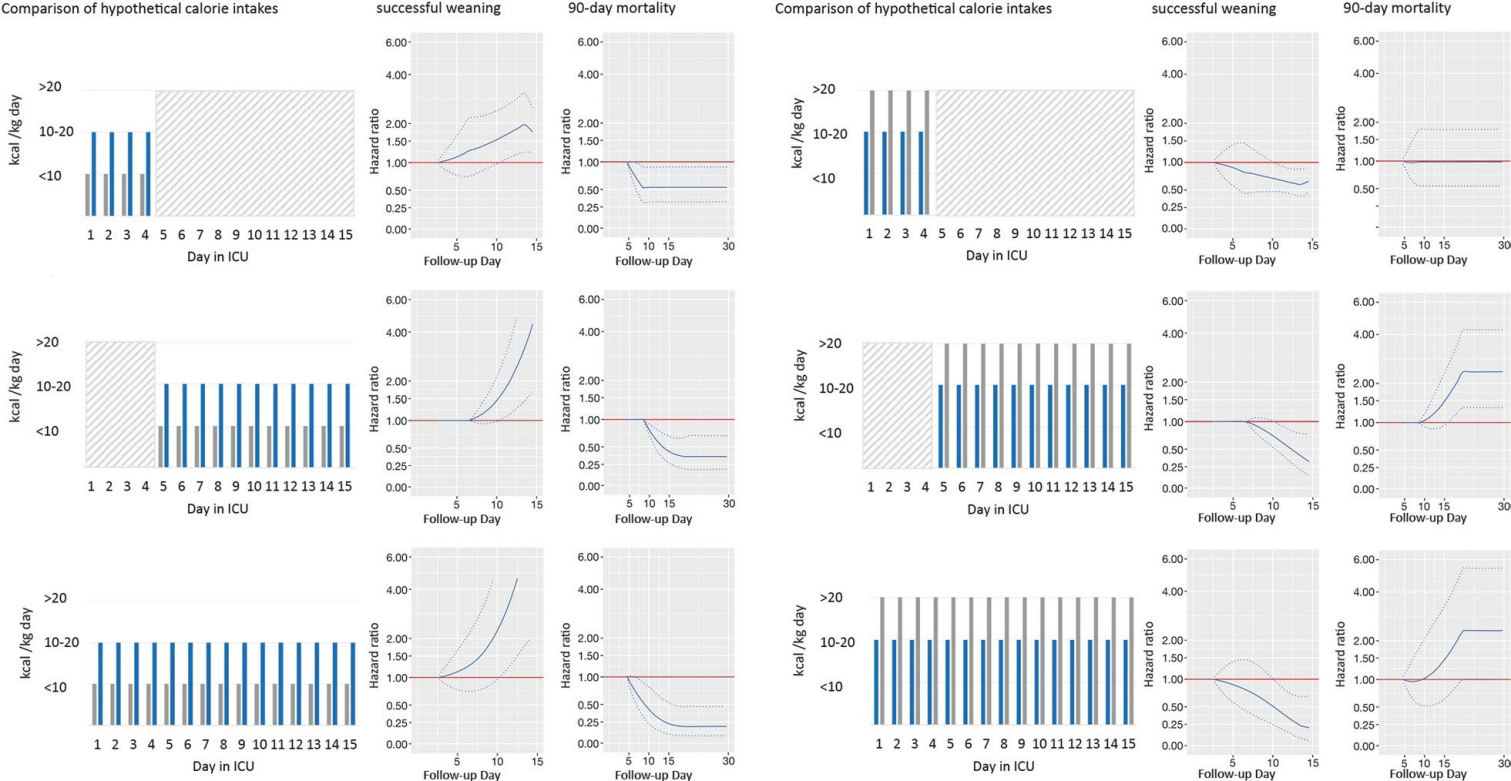
Confounder-adjusted, time-varying association of a medical nutrition therapy providing fewer vs more calories, with outcomes. Columns 1 and 4: Hypothetical medical nutrition therapy comparisons analyzing different levels of daily calorie intakes: low: < 10 kcal/kg; moderate: 10–20 kcal/kg; high: > 20 kcal/kg (Table 1). Columns 2 and 3, and 5 and 6: Corresponding time-varying associations of different hypothetical medical nutrition therapies with the hazard of successful weaning from invasive mechanical ventilation (IMV), or 90-day mortality. Gray areas indicate days with an identical calorie intake. Due to specifications of the model, this intake could have been at any intake level. Solid lines indicate hazard ratios (HR), and hatched lines indicate corresponding 95% confidence intervals (CI). Reference medical nutrition therapy is the one which provides fewer calories [e.g., a HR (and 95% CI) < 1 would indicate a longer survival time but also a longer time until extubation associated with the medical nutrition therapy providing more calories]. Please note that HRs (and corresponding 95% CIs) must be 1 for the first 2 days for IMV and 4 days for survival due to the specification of the lag time, and also for time intervals, in which calorie intake was identical within the relevant time window that affects the hazard. From the 90-day survival analysis, the HRs for the first 30 days are displayed due to estimation stability as the majority of deaths occurred until this day

**Fig. 4:**
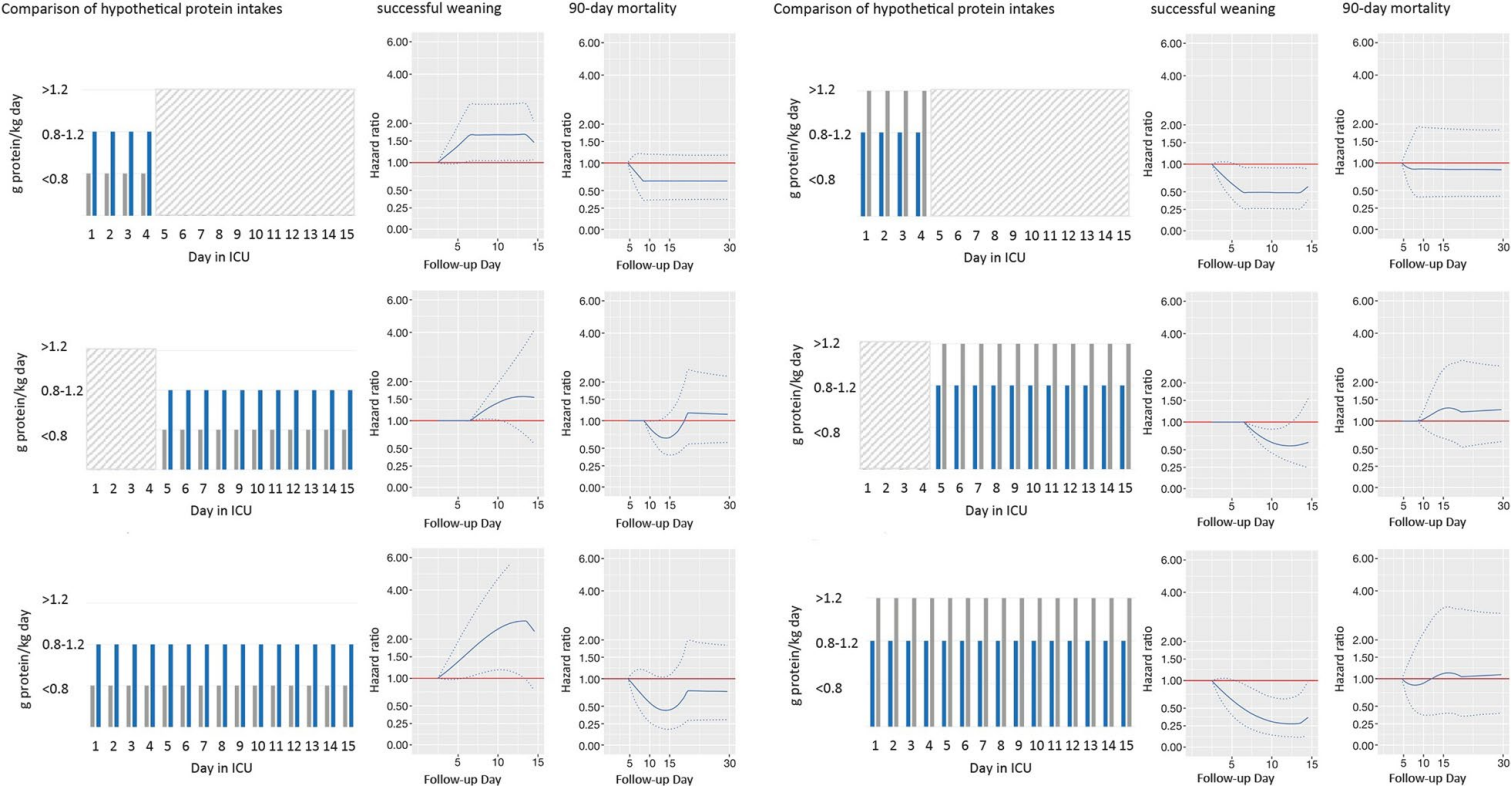
Confounder-adjusted, time-varying association of a medical nutrition therapy providing fewer vs more protein, with outcomes. Columns 1 and 4: Hypothetical medical nutrition therapy comparisons analyzing different levels of daily protein intake: low: < 0.8 g/kg; moderate: 0.8–1.2 g/kg; high: > 1.2 g/kg (Table 1). Columns 2 and 3, and 5 and 6: Corresponding time-varying associations of different hypothetical medical nutrition therapies with the hazard of successful weaning from invasive mechanical ventilation (IMV), or 90-day mortality. Gray areas indicate days with an identical protein intake. Due to specifications of the model, this intake could have been at any intake level. Solid lines indicate hazard ratios (HR), and hatched lines indicate corresponding 95% confidence intervals (CI). Reference medical nutrition therapy is the one which provides fewer protein [e.g., a HR (and 95% CI) < 1 would indicate a longer survival time but also a longer time until extubation associated with the medical nutrition therapy providing more protein]. Please note that HRs (and corresponding 95% CIs) must be 1 for the first 2 days for IMV and 4 days for survival due to the specification of the lag time, and also for time intervals, in which protein intake was identical within the relevant time window that affects the hazard. From the 90-day survival analysis, the HRs for the first 30 days are displayed due to estimation stability as the majority of deaths occurred until this day

To visualize the confounder-adjusted associations of hypothetical medical nutrition therapies with our outcomes (time to weaning from IMV and survival time), we compared a time-varying moderate intake (feeding of 10–20 kcal/kg or 0.8–1.2 g protein/kg) with a constant low intake (feeding of < 10 kcal/kg or < 0.8 g protein/kg) (Figs. 3, 4, columns 1–3). Compared to less calories, and irrespective from timing, providing 10–20 kcal/kg per day was associated with a significantly longer survival time (minimum HR 0.15 [95% CI: 0.05;0.39] on day 19, Fig. 3, rows 1 to 3) and shorter time on IMV (maximum HR 4.59 [95% CI: 1.5;14.09] on day 12, Fig. 3, row 1 to 3). This significant association was particularly evident when the difference in calorie supply was present after day 5 (for survival: minimum HR 0.30 [95% CI: 0.15;0.59] on day 19, Fig. 3, row 2; for weaning: maximum HR 4.45 [95% CI: 1.68;11.77] on day 14, Fig. 3, row 2).

Associations for protein intake were weaker, with only a moderate protein intake from day 1 to day 15 significantly associated with earlier weaning from IMV (maximum HR 2.60 [95% CI: 1.09;6.23] on day 12, Fig. 4, row 3), but not with survival. Comparison of a time-varying high intake (feeding of > 20 kcal/kg or > 1.2 g protein/kg) with a constant moderate intake (Figs. 3, 4, columns 4–6) revealed that, irrespective from timing, high calorie and protein intakes were both associated with a longer time until extubation (for calories: minimum HR 0.21 [95% CI: 0.06;0.69] on day 14, Fig. 3, row 3, column 5; for protein: minimum HR 0.28 [95% CI: 0.12;0.65] on day 11, Fig. 4, row 3, column 5), but not with a shorter survival time.

Most patients were independently mobile before ICU admission, while IMS values were lower on days 15 and 30, yet, returned to baseline by day 90 (Table 2). At all timepoints, patients still in hospital had a lower median IMS value than discharged patients (Additional file 1: Annex 1, Additional file 1: Fig. S6). Confounder-adjusted associations between nutrition and IMS values were not analyzed because of limitations from both a substantial ceiling and floor effect (Additional file 1: Annex 1, Additional file 1: Table S3).

## Discussion

To our knowledge, this is one of the largest prospective studies providing real-world evidence about nutrition and its associations with clinical outcomes in a mixed population of critically ill adult patients treated in European ICUs during a minimum ICU LOS of 5 days. Associations were assessed using a novel combination of established statistical techniques which considered time dependency of medical nutrition therapy effects, and interferences from confounding by indication [22, 23].

Median calorie and protein intake during the first 15 days after ICU admission was 15.9 [10.8;21.2] kcal/kg and 0.7 [0.4;1.0] g/kg per day, respectively. Interestingly, these intakes are very similar to observations (14.3 kcal/kg day, 0.7 g protein/kg day) made about ten years ago in a comparable cohort of international and European critically ill patients which had been included into the International Nutrition Survey [4, 24]. Similar to this longitudinal survey, we also identified a ramp-up of intakes reaching a plateau about one week after ICU admission. This shows that the current clinical practice is already following the recently published recommendations made by a group of experts in critical care nutrition with the intention to provide practical tips in complement to the active ESPEN guideline [25].

It should be noted that, on the one hand, our average calorie intake was lower than that found by the recent, large European nutritionDay ICU initiative (about 21 kcal/kg day) [26] and Latin American Screening Day (about 24 kcal/kg day) [5]. This discrepancy may be explained by a different study design. In contrast to our longitudinal study, both initiatives used a point-prevalence design favoring the inclusion of critically ill patients treated on the ICU beyond day 15 after ICU admission. On the other hand, adequacy of calorie intake (about 83%) was higher than in previous studies because a) we compared intakes to targets which were lower early after ICU admission (10–15 kcal/kg per day on days 1 to 3), and increased only thereafter, and b) we excluded patients with a short ICU LOS (< 5 days) often receiving low amounts of nutrition.

Analysis of independent determinants of macronutrient intake in our cohort revealed that specific comorbidities (especially respiratory diseases requiring invasive ventilation) predicted a higher intake, whereas intake was lower if there had been a regular assessment of nutritional needs. This observation appears to contradict results of the International Nutrition Survey where the presence of a feeding protocol at the site level was associated with higher intakes [4]. Feeding protocols communicated at the time of the International Nutrition Survey clearly aimed at the provision of more enteral calories in the light of a high target (24 kcal/kg day in the acute phase). Current assessment of individual nutritional needs, however, may have more likely considered the phase of the disease favoring lower intakes during the acute phase.

A major finding of our study was that intensivists had provided moderate amounts that were associated with the best outcomes. Optimal, phase-dependent targets for calorie and protein intake have been subject to an intense discussion throughout more than two decades, largely steered by the conflicting evidence from observational studies. A specific limiting effect was the extremely varying design, patient selection and analytical approaches of clinical studies giving room to different interpretations [27]. Limitations of observational studies result from an inadequate ratio between the number of events and confounding variables, and from the ignorance of confounding by indication, competing risks, time dependency of macronutrient intake, and time-variation/nonlinearity of associations, causing a considerable bias [28, 29]. Only recently, several randomized studies provided a clearer picture of an optimal medical nutrition therapy especially in terms of calorie intake. Meta-analyses on this subject have been reviewed [30], suggesting that calorie intake either is unimportant for outcomes, or that there might be a U-shaped relationship between calorie intake and mortality or morbidity (minimum with a moderate calorie intake), which is in line with our findings. Our data therefore support the harms associated with underfeeding and overfeeding, yet a universally accepted mechanism of action has not been established.

Quality of evidence for recommendations on protein intake is still low because of a lack of specific randomized studies. To overcome this knowledge gap, some authors extracted protein intake from large, randomized studies originally designed to study the effects of a different calorie intake. Two recent meta-analyses found that protein intakes between 0.7 and 1.3 g/kg day were unimportant for morbidity or mortality [3, 31] and a post hoc analysis of the PermiT trial [32] revealed similar results. Our results suggest a U-shaped relationship between protein and time until weaning, but not mortality. Compared to lower or higher intakes, an intake between 0.8 and 1.2 g/kg per day was associated with a shorter time until extubation, particularly if provided throughout the whole observation period. These findings suggest that nutrition strategies enabling a moderate calorie supply of 10–20 kcal/kg per day and moderate protein intake up to ~ 1.2 g/kg per day early after ICU admission have the potential to improve patient care.

The impact of nutrition on the functional recovery of critically ill patients remains unclear. Prior studies have shown inconsistent effects, presumably due to variabilities in the assessment of muscle function, different follow-up times, large drop-out rates, and, in some cases, difficulties to obtain data post ICU/hospital discharge [12, 29, 33–35]. In our study, the associations of macronutrient intakes with IMS values could not be assessed because of a ceiling effect, not allowing to identify differences in IMS values in patients able to walk independently.

### Strengths and limitations

The main strength of this study is the prospective design guaranteeing a high data quality, and the large number of patients. Our study reflects the current clinical practice of medical nutrition therapy in a diverse medical/surgical population of moderately severe, long-staying critically ill patients in European ICUs. Furthermore, for all patients, survival was followed up over 90 days after ICU admission.

This study is mainly limited by its observational nature, only allowing to assess associations between macronutrient intake and clinical outcomes, which may still be affected by potential unmeasured confounders despite the robust methodology used with respect to confounding by indication. In contrast to preceding observational studies, we considered the time dependency of macronutrient intake, and we tried to minimize interferences from a better nutritional tolerance prior to discharge/extubation and from a worse tolerance prior to death. By adjusting to oral/enteral calorie intake (in % of total intake) during the first five days of ICU stay, we directly accounted for interferences from gastrointestinal function immediately after the insult, being presumably better with oral/enteral, and worse with parenteral feeding. Our results suggest that these strategies were effective: If confounding by indication had been strong, we would have observed significant associations between an increasing intake during the acute phase and a progressively better outcome, which was not the case. Selection bias might have resulted from the nutrition-oriented nature of the study, encouraging a higher participation of sites interested in nutrition. Finally, a bias might arise from including patients in our analysis up to a BMI of 45 kg/m^2^. According to recent results, however, associations between hypothetical diets and outcomes in patients with a BMI > 30 kg/m^2^ are qualitatively comparable to those with a lower BMI [23].

## Conclusion

This prospective multinational cohort study in critically ill patients staying more than 5 days in the ICU showed that median calorie intake was slightly below the recommend target of 20–25 kcal/kg of the 2019 ESPEN guideline, whereas protein intake was clearly below the 2019 recommendation of 1.3 g/kg. Outcome analyses showed that moderate daily macronutrient intake of 10–20 kcal/kg and 0.8–1.2 g protein/kg, both approaching currently recommended targets [25], was associated with earlier weaning from IMV, and, for calories, with longer survival compared to a daily intake above or below these moderate intakes.

## Supplementary Information

**Additional file 1.** The concept of the statistical method, additional tables and figures including a description of how to interpret the pairwise comparisons.

## Data Availability

The datasets generated and analyzed during the current study are property of the study sponsor and can be requested for reasonable research purposes.

## Abbreviations

APACHE II: Acute Physiology and Chronic Health Evaluation score, second version
BMI: Body mass index
BW: Body weight
EN: Enteral nutrition
ESPEN: European Society for Clinical Nutrition and Metabolism (previously known as European Society of Parenteral and Enteral Nutrition)
HR: Hazard ratio
ICU: Intensive care unit
IMS: ICU mobility score
IMV: Invasive mechanical ventilation
LOS: Length of stay
ON: Oral nutrition
ONS: Oral nutrition supplements
PN: Parenteral nutrition
RCT: Randomized, controlled trial

## References

[1] Singer P, Blaser AR, Berger MM, Alhazzani W, Calder PC, Casaer MP (2019). ESPEN guideline on clinical nutrition in the intensive care unit. Clin Nutr.

[2] Singer P, Berger M, van den Berghe G, Biolo G, Calder P, Forbes A, Griffiths R, Kreyman G, Leverve X, Pichard C (2009). ESPEN guidelines on parenteral nutrition: intensive care. Clin Nutr.

[3] Lee Z-Y, Yap CSL, Hasan MS, Engkasan JP, Barakatun-Nisak MY, Day AG (2021). The effect of higher versus lower protein delivery in critically ill patients: a systematic review and meta-analysis of randomized controlled trials. Crit Care.

[4] Heyland DK, Dhaliwal R, Wang M, Day AG (2015). The prevalence of iatrogenic underfeeding in the nutritionally ‘at-risk’ critically ill patient: results of an international, multicenter, prospective study. Clin Nutr.

[5] Vallejo KP, Martínez CM, Adames AAM, Fuchs-Tarlovsky V, Nogales GCC, Paz RER (2017). Current clinical nutrition practices in critically ill patients in Latin America: a multinational observational study. Crit Care.

[6] Arabi YM, Aldawood AS, Haddad SH, Al-Dorzi HM, Tamim HM, Jones G (2015). Permissive underfeeding or standard enteral feeding in critically ill adults. N Engl J Med.

[7] Zusman O, Theilla M, Cohen J, Kagan I, Bendavid I, Singer P (2016). Resting energy expenditure, calorie and protein consumption in critically ill patients: a retrospective cohort study. Crit Care.

[8] Bendavid I, Zusman O, Kagan I, Theilla M, Cohen J, Singer P (2019). Early administration of protein in critically ill patients: a retrospective cohort study. Nutrients.

[9] Singer P, Anbar R, Cohen J, Shapiro H, Shalita-Chesner M, Lev S (2011). The tight calorie control study (TICACOS): a prospective, randomized, controlled pilot study of nutritional support in critically ill patients. Intensive Care Med.

[10] Heidegger CP, Berger MM, Graf S, Zingg W, Darmon P, Costanza MC (2013). Optimisation of energy provision with supplemental parenteral nutrition in critically ill patients: a randomised controlled clinical trial. The Lancet.

[11] Weijs PJ, Stapel SN, de Groot SD, Driessen RH, de Jong E, Girbes AR (2012). Optimal protein and energy nutrition decreases mortality in mechanically ventilated, critically ill patients: a prospective observational cohort study. J Parenter Enter Nutr.

[12] Compher C, Chittams J, Sammarco T, Nicolo M, Heyland DK (2017). Greater protein and energy intake may be associated with improved mortality in higher risk critically ill patients: a multicenter, multinational observational study. Crit Care Med.

[13] Allingstrup MJ, Kondrup J, Wiis J, Claudius C, Pedersen UG, Hein-Rasmussen R (2017). Early goal-directed nutrition versus standard of care in adult intensive care patients: the single-centre, randomised, outcome assessor-blinded EAT-ICU trial. Intensive Care Med.

[14] Koekkoek WACK, van Setten CHC, Olthof LE, Kars JCNH, van Zanten ARH (2019). Timing of PROTein INtake and clinical outcomes of adult critically ill patients on prolonged mechanical VENTilation: the PROTINVENT retrospective study. Clin Nutr.

[15] Hiesmayr M, Csomos A, Dams K, Elke G, Hartl W, Huet O (2021). Protocol for a prospective cohort study on the use of clinical nutrition and assessment of long-term clinical and functional outcomes in critically ill adult patients. Clin Nutr ESPEN.

[16] Tipping CJ, Bailey MJ, Bellomo R, Berney S, Buhr H, Denehy L (2016). The ICU mobility scale has construct and predictive validity and is responsive. A multicenter observational study. Ann Am Thorac Soc.

[17] Peterson CM, Thomas DM, Blackburn GL, Heymsfield SB (2016). Universal equation for estimating ideal body weight and body weight at any BMI. Am J Clin Nutr.

[18] Verreijen AM, Engberink MF, Houston DK, Brouwer IA, Cawthon PM, Newman AB (2019). Dietary protein intake is not associated with 5-y change in mid-thigh muscle cross-sectional area by computed tomography in older adults: the health, aging, and body composition (Health ABC) study. Am J Clin Nutr.

[19] Brandi LS, Santini L, Bertolini R, Malacarne P, Casagli S, Baraglia AM (1999). Energy expenditure and severity of injury and illness indices in multiple trauma patients. Crit Care Med.

[20] Choi EY, Park DA, Park J (2015). Calorie intake of enteral nutrition and clinical outcomes in acutely critically ill patients: a meta-analysis of randomized controlled trials. J Parenter Enter Nutr.

[21] Tian F, Wang X, Gao X, Wan X, Wu C, Zhang L (2015). Effect of initial calorie intake via enteral nutrition in critical illness: a meta-analysis of randomised controlled trials. Crit Care.

[22] Hartl WH, Bender A, Scheipl F, Kuppinger D, Day AG, Küchenhoff H (2019). Calorie intake and short-term survival of critically ill patients. Clin Nutr.

[23] Hartl WH, Kopper P, Bender A, Scheipl F, Day AG, Elke G (2022). Protein intake and outcome of critically ill patients: analysis of a large international database using piece-wise exponential additive mixed models. Crit Care.

[24] Ridley EJ, Peake SL, Jarvis M, Deane AM, Lange K, Davies AR (2018). Nutrition therapy in Australia and New Zealand intensive care units: an international comparison study. J Parenter Enter Nutr.

[25] Preiser J-C, Arabi YM, Berger MM, Casaer M, McClave S, Montejo-González JC (2021). A guide to enteral nutrition in intensive care units: 10 expert tips for the daily practice. Crit Care.

[26] Bendavid I, Singer P, Theilla M, Themessl-Huber M, Sulz I, Mouhieddine M (2017). NutritionDay ICU: a 7 year worldwide prevalence study of nutrition practice in intensive care. Clin Nutr.

[27] Ingels C, Langouche L, Dubois J, Derese I, Vander Perre S, Wouters PJ (2021). C-reactive protein rise in response to macronutrient deficit early in critical illness: sign of inflammation or mediator of infection prevention and recovery. Intensive Care Med.

[28] Arabi YM, Casaer MP, Chapman M, Heyland DK, Ichai C, Marik PE (2017). The intensive care medicine research agenda in nutrition and metabolism. Intensive Care Med.

[29] Rougier L, Preiser JC, Fadeur M, Verbrugge AM, Paquot N, Ledoux D (2021). Nutrition during critical care: an audit on actual energy and protein intakes. J Parenter Enter Nutr.

[30] Elke G, Hartl WH, Kreymann KG, Adolph M, Felbinger TW, Graf T (2019). Clinical nutrition in critical care medicine—guideline of the German Society for Nutritional Medicine (DGEM). Clin Nutr ESPEN.

[31] Davies ML, Chapple L-AS, Chapman MJ, Moran JL, Peake SL (2017). Protein delivery and clinical outcomes in the critically ill: a systematic review and meta-analysis. Crit Care Resusc.

[32] Arabi Y, Al-Dorzi H, Mehta S, Tamim H, Haddad S, Jones G (2018). Association of protein intake with the outcomes of critically ill patients: a post hoc analysis of the PermiT trial. Am J Clin Nutr.

[33] Fetterplace K, Deane AM, Tierney A, Beach LJ, Knight LD, Presneill J (2018). Targeted full energy and protein delivery in critically ill patients: a pilot randomized controlled trial (FEED trial). J Parenter Enter Nutr.

[34] Ridley EJ, Davies AR, Parke R, Bailey M, McArthur C, Gillanders L (2018). Supplemental parenteral nutrition versus usual care in critically ill adults: a pilot randomized controlled study. Crit Care.

[35] Dresen E, Weißbrich C, Fimmers R, Putensen C, Stehle P (2021). Medical high-protein nutrition therapy and loss of muscle mass in adult ICU patients: a randomized controlled trial. Clin Nutr.

