# Supplementary material for: Medical nutrition therapy and clinical outcomes in critically ill adults: a European multinational, prospective observational cohort study (EuroPN)
Source: Crit Care. 2022 May 18;26:143. doi: 10.1186/s13054-022-03997-z (PMC9115983; doi:10.1186/s13054-022-03997-z)
Supplement: Supplementary file 1 — Additional file 1. The concept of the statistical method, additional tables and figures including a description of how to interpret the pairwise comparisons. [file 13054_2022_3997_MOESM1_ESM.docx]

**SUPPLEMENTARY INFORMATION**

**Study title:**

Medical nutrition therapy and clinical outcomes in critically ill adults: A European multinational, prospective observational cohort study (EuroPN)

**Authors:**

Matejovic M, Huet O, Dams K, Elke G, Vaquerizo C, Csomos A, Krzych LJ, Tetamo R, Puthucheary ZA, Rooyackers O, Tjäder I, Kuechenhoff H, Hartl W, Hiesmayr M.

**Corresponding authors:**

Wolfgang H. Hartl, MD. Klinik fuer Allgemeine, Viszeral-, und Transplantationschirurgie, Klinikum der Universitaet, Campus Grosshadern, Ludwig-Maximilians-Universität Muenchen, Marchioninistraße 15, 81377 Munich, Germany.

E-mail: [whartl@med.uni-muenchen.de](mailto:whartl@med.uni-muenchen.de)

Michael Hiesmayr, MD. Division of Cardiac, Thoracic, Vascular Anesthesia and Intensive Care, and Center for Medical Statistics, Informatics and Intelligent Systems, Medical University Vienna, Spitalgasse 23, Vienna, Austria.

Email: michael.hiesmayr@meduniwien.ac.at

**Annex 1. Concept of the statistical methods**

For descriptive statistics, data are given as counts and continuous data are presented as median and interquartile range [Q1;Q3] values. Independent predictors for daily calorie and protein intakes were identified using linear mixed-effect models with repeated measures and reported as estimates and 95% CI. Study site was included as random effect, and ICU day as fixed effect. Discharge date was excluded since mostly partial nutrition data was available depending on discharge time. Continuous variables were modelled by flexible penalized spline to account for possible nonlinear relationships with the outcome variables. A p-value <0.05 was considered a significant association.

The calculation of the associations of medical nutrition therapy with time to weaning from IMV and with overall survival (up to day 90 after ICU admission) was based on a complex survival model, where the hazard is a piecewise constant function depending on the calorie intake and confounder variables, as previously introduced by Bender et al. 2018, and Hartl et al. 2019 (1, 2). In order to analyze the impact of both the timing and the time–dependent substrate intake, this novel approach allows for the estimation of cumulative effects and time-varying effects on hazards.

The confounder model was defined based on substantive and methodological considerations, resulting in a flexible nonlinear model with partially time-varying and partially time-constant associations (1-5). Nonlinear associations were estimated using penalized splines. The analysis on time to weaning was based on a subgroup of patients receiving invasive mechanical ventilation between day 1 and day 3 of ICU admission. Death while intubated was defined as a competing event in the time-to-weaning analysis (competing risk analysis)(5).

We included a random effect for the ICUs in the model to account for heterogeneity of the different ICUs. Except for nutrition variables, predictors were cumulated up to day 5 after ICU admission and were included as time-constant covariates in the confounder model. Subsequently, we modeled the association of the nutritional variables with the risk of weaning/dying by adding those variables to the final confounder model. To assess the association between nutrition and weaning or survival we used a time lag of 2 and 4 days, respectively, (*lag-time*) to minimize endogeneity (confounding by indication), and to account for treatment time required until effects of nutritional therapies on outcome might be seen. In addition, we used a *lead time* which should account for the fact that a short duration of medical nutrition therapy is unlikely to affect outcome throughout prolonged periods of time. These lag and lead times constituted a time window in which substrate intake on a specific day of medical nutrition therapy could have affected subsequent hazards of time until death or extubation. (dynamic lead time with a maximum of 12 days for weaning, and a maximum of 44 days for survival).

For the modelling of the calorie intake, level I of calorie intake (<10 kcal/kg) was defined as the reference category. Associations of levels II and III with outcome were modelled as bivariate smooth functions (smoothly time-varying effects of time-dependent covariates) that depict partial associations between substrate intake on nutrition days 1 to 15, and the chance of extubation /risk of dying during the follow up.

To assess the overall association of nutrition with weaning/mortality risk for a specific interval of the follow up, these partial associations of levels II or III were added up. The specific partial associations that were cumulated for each interval of the follow-up are circumscribed by the lag-time of two and four days, respectively. For example, mortality risk on day 13 after ICU admission was assumed to be associated only with the partial nutrition effects of nutrition days 1 to 9.

*Interval structure and Lag specification*

For the implementation of the method, we discretized the time-scale into days (24h periods) after ICU admission (see Table S1). The following aspects were important for the complex modeling of the association between substrate intake and the hazard rate: a) The model should allow the association between a certain level of substrate intake and the outcome to be cumulative over the relevant period of exposure as well as potentially time-varying; b) we assumed that substrate intake on a certain nutrition day t_e_ can only be associated with individual hazard rates after a certain lag time (t_lag_), i.e., calorie intake on nutrition day $t_{e}$ is associated with the chance of extubation /risk of dying within a certain time window during the follow up, and is not associated with that risk outside that time window.

To facilitate the interpretation of these time-varying hazard ratios (HR) between nutrition and outcome, we constructed 6 pairwise comparisons of hypothetical medical nutrition therapies, reflecting different time-varying calorie and protein intakes over days 1-15, thereby differentiating between an early (day 1 to 4) and a late (after day 4) period.

Daily nutrient intake per kg BW was therefore classified using established thresholds for calories and protein, respectively: low: <10 kcal/kg, <0.8 g/kg; moderate: 10-20 kcal/kg, 0.8-1.2 g/kg; high: >20 kcal/kg, >1.2 g/kg.

For reader´s interpretation, a detailed explanation on the translation of these pairwise comparisons into the cox-type models are provided in this eSupplement (eFigure 1).

R packages pammtools (4) and mgcv (6) were used to estimate corresponding models.

**eTable 1.**

Association between confounders and time to weaning from invasive mechanical ventilation (cox-type model for protein intake)

|  | **Variable name** | **HR** | **LL CI 95%** | **UL CI 95%** | **p-value** |
| --- | --- | --- | --- | --- | --- |
| Predictor variables | Age | 1.00 | 0.99 | 1.00 | 0.233 |
|  | Female vs. male | 1.20 | 0.96 | 1.51 | 0.117 |
|  | Body weight (kg) | 1.00 | 0.99 | 1.00 | 0.181 |
|  | Surgical vs. non-surgical ICU admission | 1.47 | 1.13 | 1.92 | 0.004 |
|  | Main reason for ICU admission: Infection (yes vs.no) | 1.27 | 1.00 | 1.61 | 0.049 |
|  | Main reason for ICU admission: Respiratory (yes vs.no) | 0.61 | 0.48 | 0.77 | <0.001 |
|  | Number of severe comorbidities | 1.14 | 1.01 | 1.29 | 0.036 |
|  | APACHE II | 1.00 | 0.99 | 1.01 | 0.724 |
|  | Average SOFA score of preceding days (d1-d5) | 0.89 | 0.85 | 0.92 | <0.001 |
|  | Sum of preceding days with HAIs (d1-d5) | 0.90 | 0.83 | 0.97 | 0.007 |
|  | Baseline functional status | 0.96 | 0.90 | 1.02 | 0.203 |
|  | % of total calories given via the enteral and oral route (d1-d5) | 1.00 | 1.00 | 1.01 | 0.091 |
| Random effect | Study site |  |  |  | <0.001 |

APACHE: Acute Physiology and Chronic Health Evaluation; ICU: Intensive care unit; HR: Hazard ratio; LL: Lower limit; UL: Upper Limit; CI: Confidence interval, HAI: hospital acquired infection

A HR < 1 indicates a longer time until extubation.

**eTable 2.**

Association between confounders and 90-day survival time (cox-type model for protein intake)

|  | **Variable name** | **HR** | **LL CI 95%** | **UL CI 95%** | **p-value** |
| --- | --- | --- | --- | --- | --- |
| Predictor variables | Age | 1.03 | 1.02 | 1.05 | <0.001 |
|  | Body weight (kg) | 0.99 | 0.98 | 1.00 | 0.055 |
|  | Surgical vs. non-surgical ICU admission | 0.65 | 0.46 | 0.90 | 0.011 |
|  | Main reason for ICU admission: Cardiac (yes vs.no) | 1.18 | 0.88 | 1.59 | 0.269 |
|  | Main reason for ICU admission: Infection (yes vs.no) | 1.23 | 0.93 | 1.64 | 0.146 |
|  | Main reason for ICU admission: Respiratory (yes vs.no) | 1.27 | 0.95 | 1.69 | 0.105 |
|  | APACHE II | 1.00 | 0.99 | 1.02 | 0.604 |
|  | Average SOFA score of preceding days (d1-d5) | 1.14 | 1.09 | 1.20 | <0.001 |
|  | Sum of preceding days with HAIs (d1-d5) | 1.04 | 1.01 | 1.07 | 0.007 |
|  | Sum of preceding days on invasive mechanical ventilation (d1-d5) | 1.01 | 0.98 | 1.04 | 0.549 |
|  | Limited medical support (yes vs. no) | 3.66 | 2.15 | 6.24 | <0.001 |
|  | % of total calories given via the enteral and oral route (d1-d5) | 1.00 | 0.99 | 1.00 | 0.149 |
| Random effect | Study site |  |  |  | <0.001 |

APACHE: Acute Physiology and Chronic Health Evaluation; ICU: Intensive care unit; HR: Hazard ratio; LL: Lower limit; UL: Upper Limit; CI: Confidence interval, HAI: hospital acquired infection

A HR > 1 indicates a shorter survival time.

**eTable 3.**

Proportion of patients with ICU Mobility (IMS) score of 0 and 10

|  | No. (%) |
| --- | --- |
| IMS before ICU admission, n=1168 |  |
| Score 0 | 73 (6.3) |
| Score 10 | 770 (65.9) |
| IMS D15, n=1057 |  |
| Score 0 | 212 (20.0) |
| Score 10 | 270 (25.5) |
| IMS D30, n=949 |  |
| Score 0 | 74 (7.8) |
| Score 10 | 383 (40.4) |
| IMS D90, n=844 |  |
| Score 0 | 9 (1.1) |
| Score 10 | 524 (62.1) |

**eFigure 1.**

Explanation of the pairwise comparison of two hypothetical medical nutrition therapies and their associations with time to weaning from invasive mechanical ventilation

1. Design of the therapy comparison.

Grey areas indicate days with an identical calorie intake. Due to specifications of the model this intake could have been at any intake level.


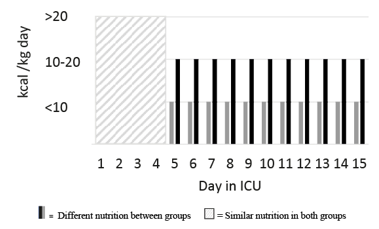


Comparison of a late (> D4) moderate with a late (> D4) low calorie intake.

1. Confounder-adjusted association between hypothetical medical nutrition therapies and time until extubation. Reference medical nutrition therapy is the one providing less calories.


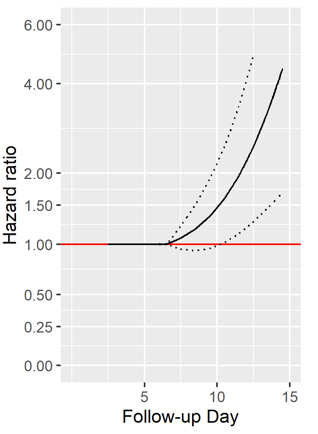


Solid lines: hazard ratios (HR), hatched lines: corresponding 95% confidence bands (CI).

Significant association when HR and 95% CI do not cross the red line (HR=1)

Lag-time two days.

For patients still requiring IMV on day 12, the chance of weaning on the following day is 3x higher in the moderate intake group compared to the low intake group.

HR=1 because the two hypothetical medical nutrition therapies did not differ for the first four days

Interpretation of hazard ratio (HR):

- - HR >1 indicates a shorter time until extubation associated with the medical nutrition therapy providing more calories
  - HR <1 indicates a longer time until extubation associated with the medical nutrition therapy providing more calories

**eFigure 2.**

Kaplan-Meier Plot showing survival rates over the study period


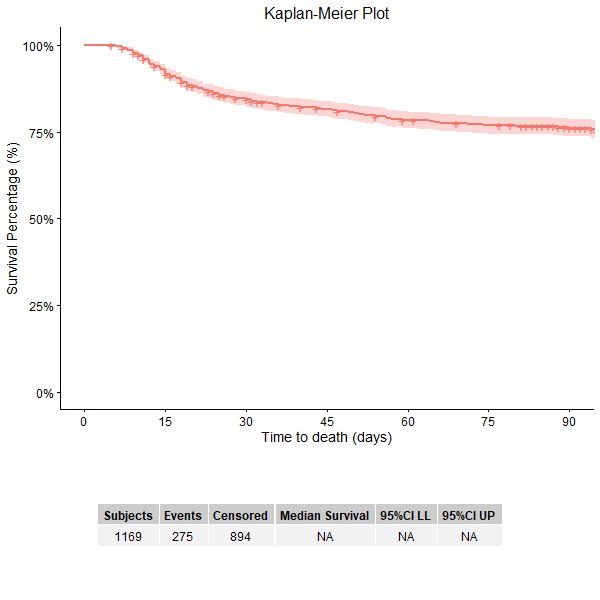


**eFigure 3.**

Cumulative incidence plot showing weaning events and deaths up to the end of the follow-up period for weaning


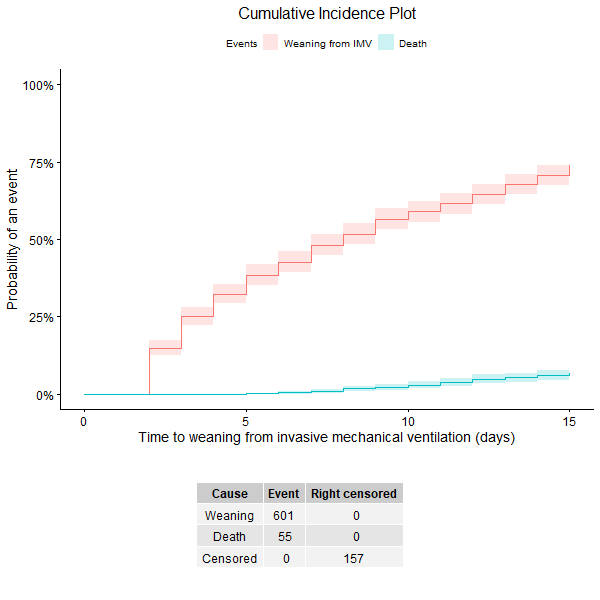


**eFigure 4.**

Daily calorie intake, excluding patients without any nutrition


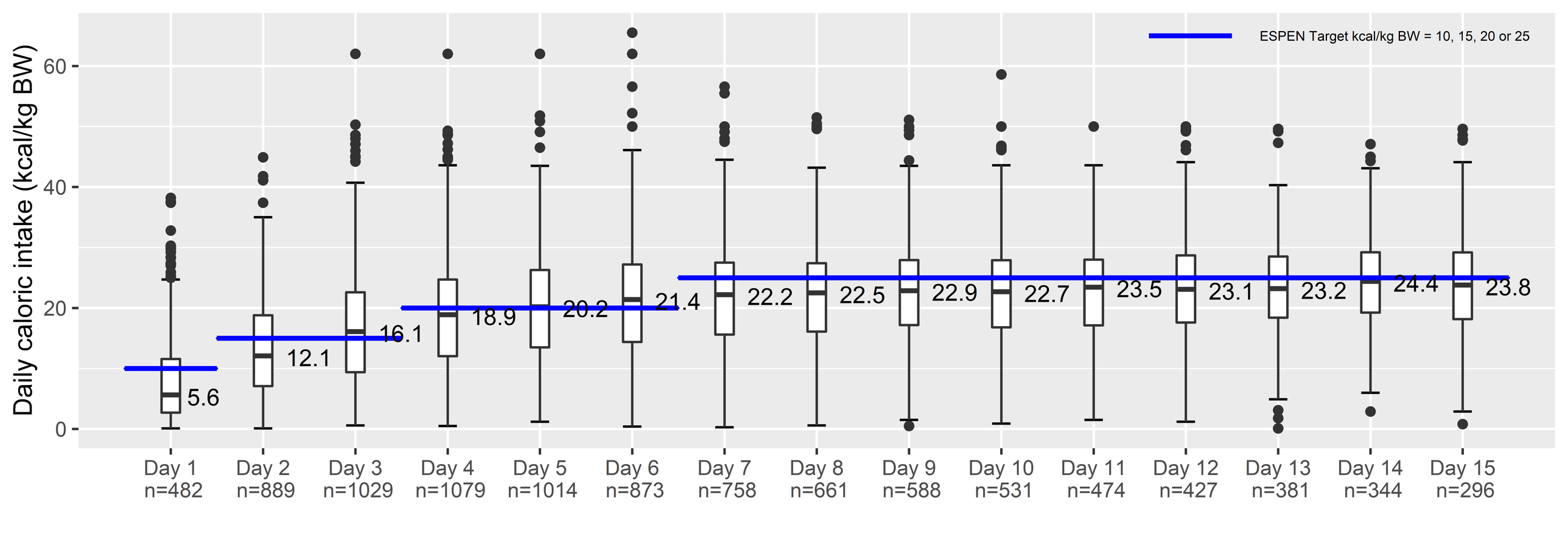


Intake is presented as median, interquartile range, minimum and maximum values with outliers versus pre-defined targets (blue horizontal bars) based on the 2019 ESPEN Guideline on clinical nutrition in critical care (7).

**eFigure 5.**

Daily protein intake, excluding patients without any nutrition


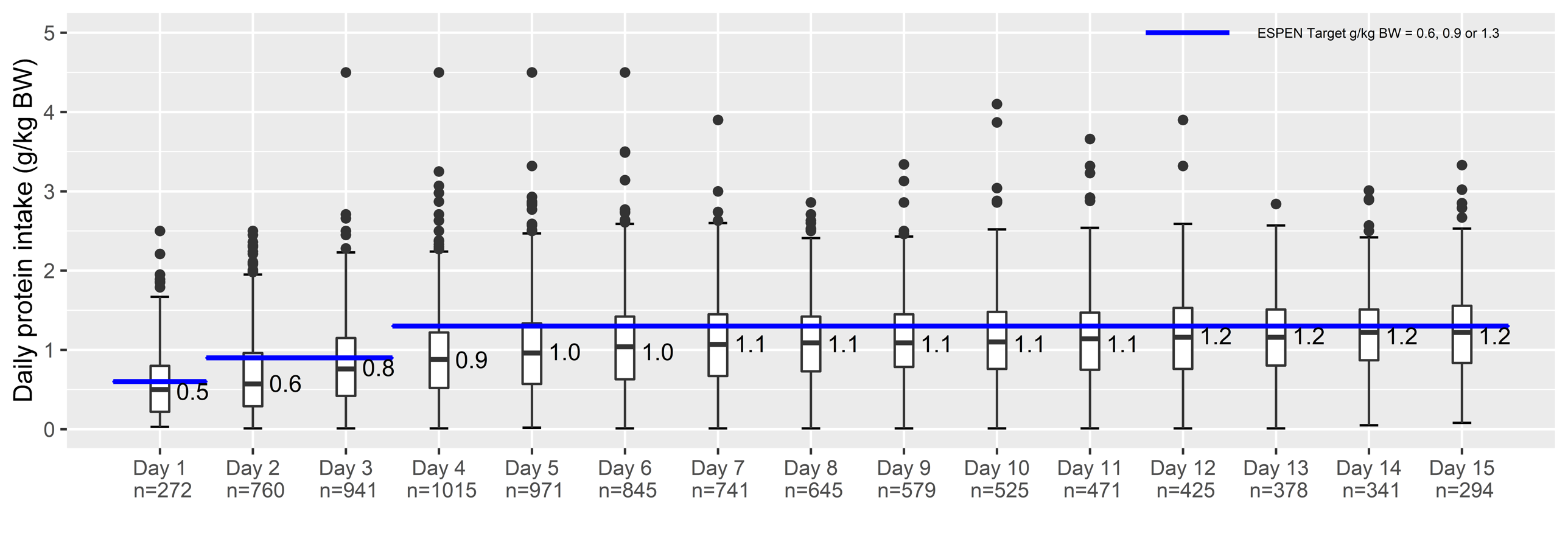


Intake is presented as median, interquartile range, minimum and maximum values with outliers versus pre-defined targets (blue horizontal bars) based on the 2019 ESPEN Guideline on clinical nutrition in critical care (7).

**eFigure 6.**

Box plots of ICU mobility (IMS) score at baseline, reflecting IMS score from before ICU admission, at days 15, 30, and 90 for patients still in hospital vs. discharged from hospital


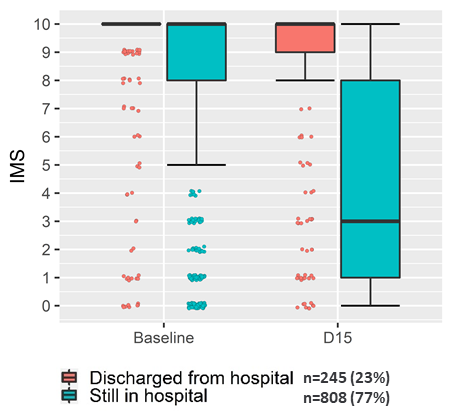


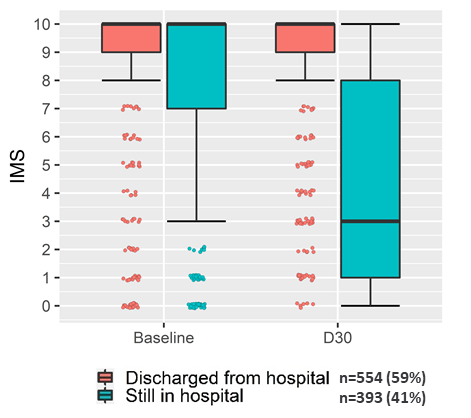


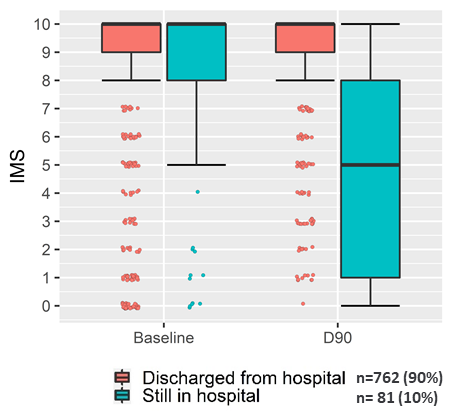


Analysis based on surviving patients with available IMS scores at respective time points.

Graph shows median, IQR, min and max with outliers.

**REFERENCES**

1. Hartl WH, Bender A, Scheipl F, Kuppinger D, Day AG, Küchenhoff H. Calorie intake and short-term survival of critically ill patients. Clinical Nutrition. 2019;38(2):660-7.

2. Bender A, Scheipl F, Hartl W, Day AG, Küchenhoff H. Penalized estimation of complex, non-linear exposure-lag-response associations. Biostatistics. 2019;20(2):315-31.

3. Bender A, Groll A, Scheipl F. A generalized additive model approach to time-to-event analysis. Statistical Modelling. 2018;18(3-4):299-321.

4. Bender A, Scheipl F. pammtools: Piece-wise exponential additive mixed modeling tools. arXiv preprint arXiv:180601042. 2018.

5. Kopper P. Flexible estimation of complex effects in the context of competing risks survival analysis: Ludwig Maximilian University; 2020. DOI: 10.5282/ubm/epub.72320

6. Wood S. Mixed GAM Computation Vehicle with Automatic Smoothness Estimation. Generalized Additive Models: An Introduction with R 2nd. ed. Boca Raton, USA: Chapman and Hall/CRC; 2017. p. 1: 8-34.

7. Singer P, Blaser AR, Berger MM, Alhazzani W, Calder PC, Casaer MP, et al. ESPEN guideline on clinical nutrition in the intensive care unit. Clinical nutrition. 2019;38(1):48-79.
